# Hypoxia-Responsive Oxygen Nanobubbles for Tissues-Targeted Delivery in Developing Tooth Germs

**DOI:** 10.3389/fcell.2021.626224

**Published:** 2021-02-15

**Authors:** Eun-Jung Kim, Ji-Eun Lee, Semi Yoon, Dong-Joon Lee, Han Ngoc Mai, Hiroko Ida-Yonemochi, Jonghoon Choi, Han-Sung Jung

**Affiliations:** ^1^Division in Anatomy and Developmental Biology, Department of Oral Biology, Taste Research Center, Oral Science Research Center, BK21 FOUR Project, Yonsei University College of Dentistry, Seoul, South Korea; ^2^School of Integrative Engineering, Chung-Ang University, Seoul, South Korea; ^3^Division of Anatomy and Cell Biology of the Hard Tissue, Department of Tissue Regeneration and Reconstruction, Niigata University Graduate School of Medical and Dental Sciences, Niigata, Japan

**Keywords:** tooth, hypomineralization, hypoxia, oxygen nanobubble, apoptosis, proliferation, metabolism

## Abstract

Hypoxia is a state of inadequate supply of oxygen. Increasing evidence indicates that a hypoxic environment is strongly associated with abnormal organ development. Oxygen nanobubbles (ONBs) are newly developed nanomaterials that can deliver oxygen to developing tissues, including hypoxic cells. However, the mechanisms through which nanobubbles recover hypoxic tissues, such as developing tooth germs remain to be identified. In this study, tooth germs were cultured in various conditions: CO_2_ chamber, hypoxic chamber, and with 20% ONBs for 3 h. The target stages were at the cap stage (all soft tissue) and bell stage (hard tissue starts to form). Hypoxic tooth germs were recovered with 20% ONBs in the media, similar to the tooth germs incubated in a CO_2_ chamber (normoxic condition). The tooth germs under hypoxic conditions underwent apoptosis both at the cap and bell stages, and ONBs rescued the damaged tooth germs in both the cap and bell stages. Using kidney transplantation for hard tissue formation *in vivo*, amelogenesis and dentinogenesis imperfecta in hypoxic conditions at the bell stage were rescued with ONBs. Furthermore, glucose uptake by tooth germs was highly upregulated under hypoxic conditions, and was restored with ONBs to normoxia levels. Our findings indicate that the strategies to make use of ONBs for efficient oxygen targeted delivery can restore cellular processes, such as cell proliferation and apoptosis, glucose uptake, and hypomineralization in hypoxic environments.

## Introduction

Tooth development is a complex process mediated through a series of signals between two adjacent tissues, the epithelium, and the underlying mesenchyme (Kim et al., 2008). Mammalian teeth form via morphogenesis of individual tooth germs, which begin as dental placodes in the embryonic oral ectoderm and sequentially pass through the bud, cap, and bell stages (Kwon et al., 2015). During morphogenesis, the epithelium is called enamel organ, which consists of the inner enamel epithelium and differentiating to enamel producing ameloblasts, the outer enamel epithelium and the stellate reticulum and stratum intermedium cells (Jussila and Thesleff, 2012). The growth and folding of the inner enamel epithelium during the bell stage determine the size and shape of the tooth crown (Yu and Klein, 2020). The shape is fixed when the organic matrix of dentin and enamel mineralize because dentin or enamel does not remodels later (Lesot and Brook, 2009).

Signals from the dental epithelium first induce the differentiation of underlying mesenchymal cells into odontoblasts (Jussila and Thesleff, 2012). The odontoblasts deposit dentin matrix and signal back to the epithelium, inducing differentiation of epithelial cells into functional ameloblasts (Karcher-Djuricic et al., 1985). Odontoblasts are responsible for the formation of dentin and pre-dentin, which is an immature mineralized tissue (Smith and Nanci, 1995). Enamel development and mineralization is a complex process that tightly regulates ameloblasts.

Enamel or dentin formation are affected by several factors, and the changes induced during enamel or dentin formation impact the enamel or dentin (Heijs et al., 2007; Padavala and Sukumaran, 2018). Developmental defects of the enamel and dentin occur in rare genetic disorders, and are termed amelogenesis imperfecta (AI) or dentinogenesis imperfecta (DGI) (Barron et al., 2008). AI describes a group of genetic conditions that result in defects in the formation of the tooth enamel. Mutations in many genes including amelogenin (AmelX for female and AMELY for males), enamelin (ENAM), and ameloblastin (Ambn), are known to cause AI, and affect the proteins secreted by enamel secreting cells, ameloblasts (Smith and Nanci, 1995; Smith et al., 2017). AI has been broadly classified into hypoplastic and hypomineralized types (Gadhia et al., 2012). Dentine encloses the dental pulp and is surrounded by the enamel. DGI and dentine dysplasia (DD) include a group of autosomal dominant genetic conditions characterized by abnormal dentine structures (Barron et al., 2008). DGI type I is inherited by osteogenesis imperfecta and recent genetic studies suggest that involvement of mutations in the genes encoding collagen type 1, COL1A1, and COL1A2. All other forms of DGI, appear to result from mutations in the gene encoding dentine sialophosphoprotein (DSPP), suggesting that these conditions are allelic. Demarcated enamel opacities that affect one or more permanent first molars, and frequently also incisors, can occur in teeth. This condition is referred to molar-incisor hypomineralization (MIH) (Alaluusua, 2010; Garg et al., 2012; Sidaly et al., 2015; Lee et al., 2020). Hypoxia has been suggested as a possible etiological factor of MIH. Although the occurrence of MIH in humans does not impact survival, it can have aesthetic effects, and cause dental defects. However, the etiology of hypomineralization during enamel or dentin formation is not fully understood.

Oxygen is the most critical molecule for survival of most organisms (Sitkovsky et al., 2004). Hypoxia is a condition of insufficient supply of oxygen to cells and tissues. Hypoxia inducible factor 1 (HIF-1α), a key regulator of hypoxia response, has complex roles. HIF-1α can induce apoptosis, prevent cell proliferation, or even stimulate cell proliferation. The severity of hypoxia determines whether cells become apoptotic or adapt to hypoxia and survive (Greijer and van der Wall, 2004; Guo, 2017).

HIF-1α initiates apoptosis by inducing high concentrations of pro-apoptotic proteins, and causes stabilization of p53 (Greijer and van der Wall, 2004). p53 plays a critical role in inducing cell cycle arrest or apoptosis (Nakamizo et al., 2008). Under normal conditions, p53 remains inactive due to its rapid degradation by the ubiquitin ligase, Mdm2 (Marine and Lozano, 2010). However, upon cellular stress, such as in hypoxic conditions, p53 becomes phosphorylated resulting in inhibition of Mdm2-mediated degradation and accumulation of transcriptionally active p53, which activates several targets including cell cycle inhibitors and pro-apoptotic proteins resulting in apoptosis or proliferation arrest (Yan et al., 2009; Sadagopan et al., 2012). Furthermore, caspases play an essential role in apoptosis (Guo, 2017). p53 and other signaling pathways lead to caspase activation, including caspase-3, that cleaves essential cellular proteins and causes apoptosis (Speidel, 2010). On the other hand, regulation by HIF-1α is closely related to energy metabolism (Rastogi et al., 2007). In response to hypoxia, cells increase glucose uptake by upregulating membranous expression of glucose transporter-1 (GLUT-1) (Malhotra and Brosius, 1999). Facilitating GLUT-1 can reduce hypoxia-induced apoptosis due to the protective role of glycolysis of glucose. During tooth development, glucose uptake mediated in GLUT-1 is essential for early tooth morphogenesis and size determination of murine molars (Ida-Yonemochi et al., 2012). However, the mechanisms through which HIF-1α, induced by hypoxic conditions, regulates tooth development and mineralization by cellular events, such as proliferation, cell apoptosis and metabolism has not been studied.

Oxygen nanobubble (ONB) is a newly developed material, which can deliver acceptable oxygen to developing tissues, including hypoxic cells (Bhandari et al., 2017; Khan et al., 2018). ONBs consist of a phospholipid hydrophilic shell with oxygen gas inside (Bhandari et al., 2017; Khan et al., 2018). ONBs release oxygen via a diffusion mechanism, as phospholipids are permeable to gas (Bhandari et al., 2017; Khan et al., 2018). ONBs released ~500 μg/mL more oxygen (Bhandari et al., 2017; Khan et al., 2018). It has been demonstrated that ONBs reverse hypoxia and suppress HIF-1α activity in tumor cells (Khan et al., 2018). This study aimed to investigate whether oxygen delivery into developing tooth germs via ONBs improves the hypoxic condition, and understand how the mineralization of enamel and dentin can be regulated using ONBs. A new hypoxia-based strategy for regenerative approaches in the dental field is presented.

## Materials and Methods

All experiments were performed according to the guidelines of the Yonsei University College of Dentistry, Intramural Animal Use and Care Committee (2019-0306).

### Animals

Adult ICR mice (purchased from Koatech Co, Pyeongtaek, Korea) were housed in a temperature-controlled room (22°C) under artificial lighting (lights on from 05:00 to 17:00) and 55% relative humidity with access to food and water *ad libitum*. Embryos were obtained from time-mated pregnant mice. E0 was designated as the day on which the presence of a vaginal plug was confirmed. Embryos from each developmental stage (E13 for cap stage, and E16 for bell stage) were used in this study.

### Preparation of Oxygen Nanobubbles (ONBs)

The ONBs were prepared by following the method reported previously (Khan et al., 2018, 2019, 2020). The mixture of DSPC and DSPE-PEG-Amine was added to a 3-neck-flask that the molar ratio of the solution was to become DSPC: DSPE-PEG-Amine = 85:15. The solution of DSPC and DSPE-PEG-Amine was then introduced to an oven set at 70°C for 2 h to remove a chloroform in the solution. Next, the 10 mL of DPBS was added to the completely dried mixture to resuspend the solute. The redissolved solution was sonicated for 10 min at 60°C that was above the transition temperature (i.e., 55°C) of DSPC and DSPE-PEG-Amine. At the same time, the solution was bubbled with an oxygen gas for 3 min. While supplying an oxygen gas to the mixture, a tip sonication was carried out to decrease the size of bubbles (on/off for 2 s each, for 5 min total, at 25°C). After the sample was filtered through a 1 μm PTFE syringe filter, it was stored in a refrigerator at 4°C before the experiments. ONB samples of other compositions were also produced in the same way as described above.

### *In vitro* Organ Culture

The lower molar tooth germs were carefully dissected from the mandibles of mouse embryos at E13 and E16. These dissected tooth germs were placed in the Trowell-type organ culture for 3 h. The hypoxic experiment and ONBs treatment experiment followed the procedure of Khan et al. (2018). Briefly, 35 mm organ-culture dishes were placed inside the hypoxic chamber during the hypoxia experiment. Argon gas was purged through the chamber for 20 min to replace the air inside the chamber. For the reversal of hypoxic condition, 20% ONB was treated on tooth germs *in vitro* culture media (10% FBS in DMEM) for 1 h after cultured in hypoxic condition.

### Luciferase Assay

Mouse HIF-1α CDS was cloned into pCMV-Myc vector (Addgene, MA, USA), promoter regions of mouse GLUT1 and GLUT2 were cloned into the pGL3 basic vector (Promega, WI, USA). Human Embryonic kidney (HEK293T) cells were grown in 60 mm cell dishes to 80% confluence before transfection with total of 3 μg each of the reporter plasmids and Myc tagged HIF-1α plasmid. The total amount of plasmid concentration used for transfection was normalized using the pCMV empty vector. The Renilla pRL-SV40 plasmid was used as a control for the transfection efficiency. FugeneFD (Promega) was used as transfection reagent. Forty-eight hours after transfection, cells were harvested, and luciferase activity in the cells was analyzed by a Dual- Luciferase Reporter Assay System (Promega).

### Immunofluorescence

The specimens were embedded in wax using conventional methods. Sections (4 μm thickness) of the specimens were boiled in 10 mM citrate buffer (pH 6.0) for 20 min and cooled at room temperature for 20 min. The specimens were incubated with primary antibodies against HIF-1α (Abcam, CAM, UK; dilution 1:200), phosphorylated p53 (Cell signaling; dilution 1:100), cleaved Caspase-3 (Cell signaling, MA, USA; dilution 1:100), PCNA (Abcam; dilution 1:200), Glut-1(Abcam; dilution 1:200), Glut-2 (Novus biologicals, CR, USA; dilution 1:200), MMP20 (Abcam; dilution 1:200), Amelx (Santa Cruz Biotechnology, TX, USA; dilution 1:200), Dmp1 (LSBio, WA, USA; dilution 1:200), Dspp (Santa Cruz Biotechnology; dilution 1:200) at 4°C overnight. The specimens were incubated with goat anti-rabbit Alexa Fluor 488 (Thermo Fisher Scientific, MA, USA; dilution 1:200), goat anti-mouse Alexa Fluor 555 (Thermo Fisher Scientific; dilution 1:200) antibodies. The sections were counterstained with DAPI (Molecular Probes, OR, USA, dilution 1:1,000) and examined using a confocal laser microscope (DMi8; Leica, Wetzlar, Germany).

### Scanning Electron Microscope

Calcified tooth, after fracture, were analyzed by SEM. Calcified teeth from kidney transplant were fixed in 4% paraformaldehyde, post-fixed with osmium, dehydrated in a series of graded ethanols, with a final immersion in 100% hexaethyldisilazane. Samples were then mounted on carbon stubs, coated with gold/palladium with a Balzar Med 10 evaporator and examined with a FE-SEM (Merlin, Zeiss) scanning electron microscope coupled with an energy-dispersion spectrometer (EDS) system.

### Statistical Analysis

GraphPad Prism software was used for statistical analysis and graphical representations of the data (GraphPad, La Jolla, CA). ANOVA were performed on the data and significance was checked. Non-significant values have been shown as ns in the results section, while ^*^, ^**^, ^***^, and ^****^ describe *p*-values of < 0.05, 0.01, 0.001, and 0.0001, respectively.

### Kidney Transplantation

The kidney was taken from the incision of dorsal side of 6-week-old ICR male mouse. The small hole at the renal capsule covering the kidney were made carefully with tungsten needle. Tooth germs cultured with each condition were inserted into the hole. The kidney were then placed back inside the abdominal cavity, and the incision was sutured. One week after the surgery, the mice were sacrificed in a CO_2_ chamber and the kidneys were taken out, and fixed in 4% paraformaldehyde and scanned using a microCT scanner. Images were reconstructed using microview software. After scanning, samples were decalcified with 10% EDTA and performed histological analyses.

### Real Time-Quantitive Polymerase Chain Reaction (RT-qPCR)

For quantification of the levels of RNA, the tooth germs under each condition were microdissected at E16 and RNA was extracted with TRIzol reagent. After DNase I treatment, the RNA was purified with an RNeasy Kit (Qiagen, Hilden, Germany). RT-qPCR was performed with a Thermal Cycler Dice™ Real Time System and SYBR Premix EX Taq™ (Takara, Kyoto, Japan) according to the manufacturer's instructions.

The primers used for amplification were as follows:

Bmp2

Forward 5′-TCAGCATGTTTGGCCTGAAGCA-3′

Reverse 5′-GCCGTTTTCCCACTCATCCTCGGAA-3′

Bmp4

Forward 5′-GACTTCGAGGCGACACTTCT-3′

Reverse 5′-ACTGGTCCCTGGGATGTTCT-3′

Bmp7

Forward 5′-ACTCCTACATGAACGCCACCAACCA-3′

Reverse 5′-TCGAAGATTTGGAAAGGTGTGGCCC-3′

Fgf2

Forward 5′-GAATTCCGGCTCTACTGCAAGAAC-3′

Reverse 5′-GAATTCTTATACTGCCCAGTTCGT-3′

Fgf10

Forward 5′-CATGTGCGGAGCTACAATCA-3′

Reverse 5′-CCCCTTCTTGTTCATGGCTA-3′

Shh

Forward 5′-GCTCTACCACATTGGCACCT-3′

Reverse 5′-TGCGCTTTCCCATCAGTT-3′

## Results

### Induction of HIF-1α and Change in Proliferation and Apoptosis in Tooth Germs at E13 Under Hypoxic Conditions

**Figure 1:**
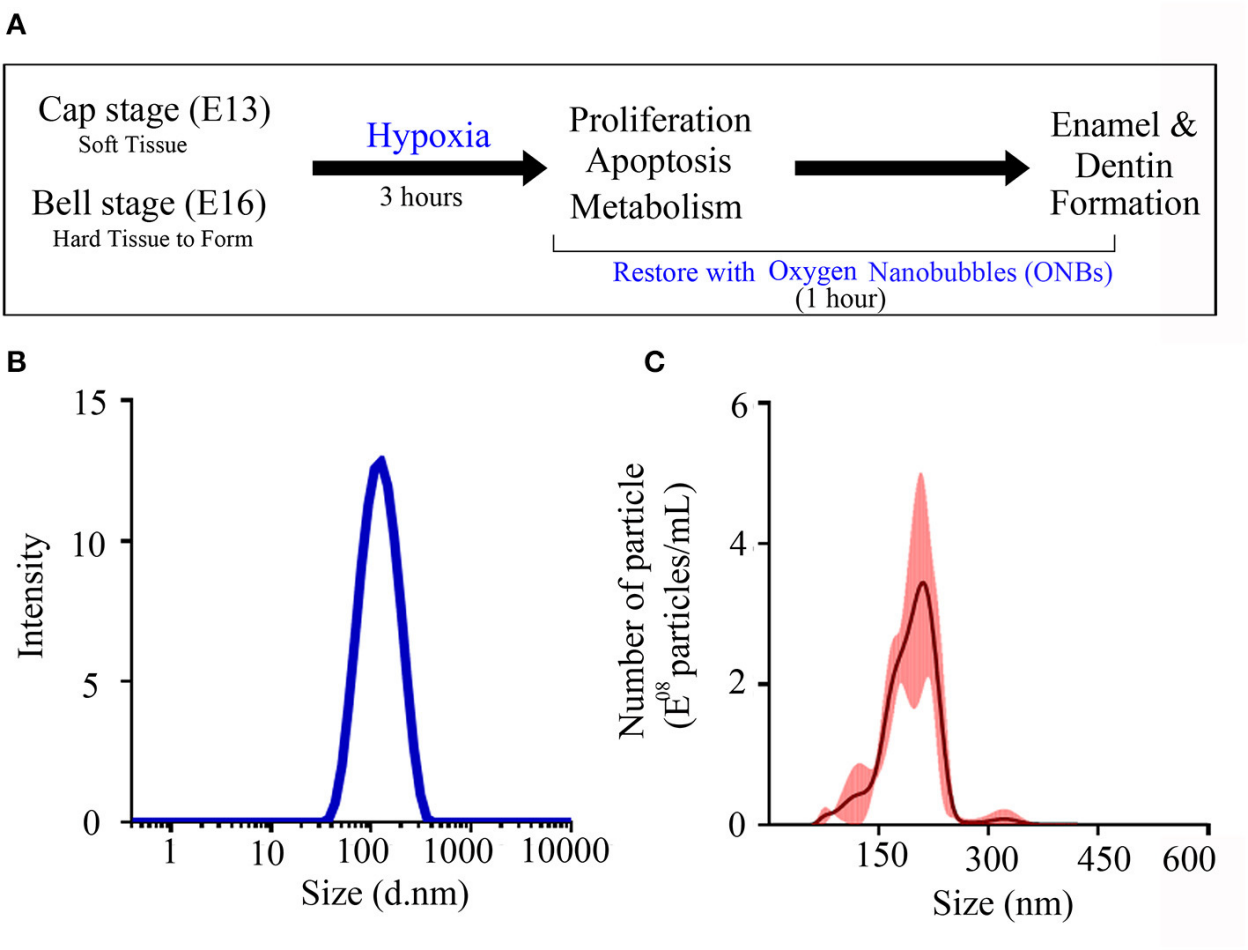
Characterization of oxygen nanobubbles. **(A)** The experiment schematic diagram. Cap stage (E13) and bell stage (E16) tooth germs were cultured in normoxia condition and hypoxia condition. These damaged tooth germs with hypoxic condition were tried to recover with oxygen nanobubbles (ONBs) for 1 h. After 3 h, *in vitro* cultured tooth germs under normoxia, hypoxia condition, and rescued by ONBs were collected, and confirmed the changes of cell proliferation, apoptosis, and metabolism. Furthermore, hard tissue including enamel and dentin were produced under the kidney capsule for 1 week and for 4 weeks. **(B,C)** The size characterization of ONBs using analytical methods. **(B)** The average particle size of ONBs obtained by a dynamic light scattering (DLS) measurement. Average diameter: 117.1 nm (±0.798 nm). **(C)** The average particle size and the enumeration of ONBs measured by a nanoparticle tracking analysis (NTA) method. Total particle concentration: 2.62E+08 particles/mL (±3.24E+10 particles/mL).

To identify the role of oxygen in enamel and dentin mineralization, tooth germs at the cap (consisting of soft tissue only) and bell (in which hard tissue begins to form) stages were cultured *in vitro* for 3 h under normoxic and hypoxic conditions (Figure 1A). Hypoxic tooth germs were incubated for 1 h in culture media containing 20% ONB to reverse the hypoxic condition. The average size of the ONB particles used in this study is 117.1 nm (±0.798 nm) (Figure 1B), and the concentration of the particles is 2.62E+08 particles/mL (±3.24E+10 particles/mL) (Figure 1C).

**Figure 2:**
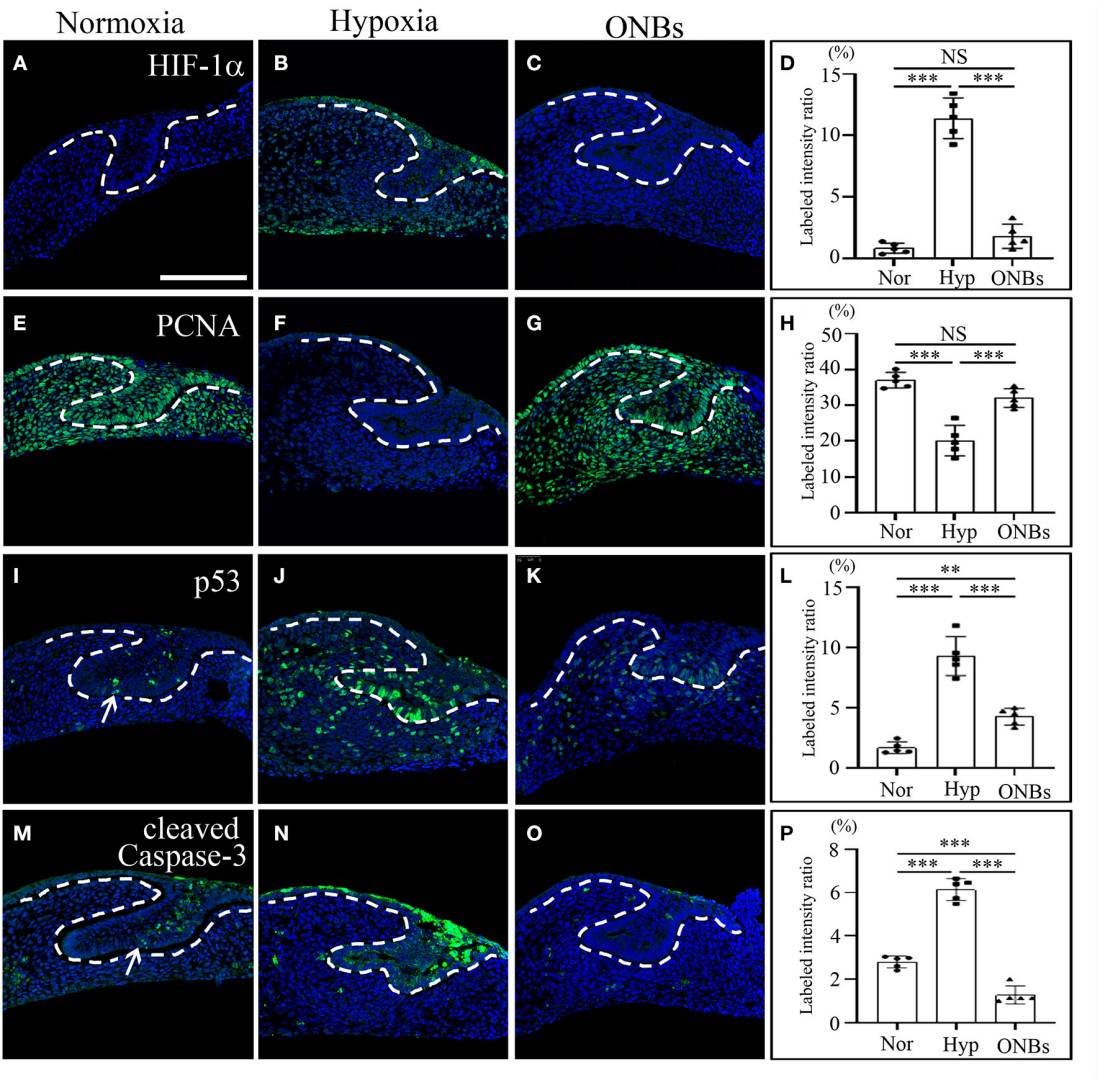
Change of cell proliferation and apoptosis at E13 tooth germs in normoxia, hypoxia, and ONB treatment. At E13, immunofluorescence was performed to show the expression change of **(A–D)** HIF-1α, **(E–H)** PCNA, **(I–L)** phosphorylated p53, **(M–P)** cleaved Caspase-3 in the frontal sections of tooth germs. HIF-1α expression is not expressed **(A)** in normoxia, increased **(B)** in hypoxia, but decreased **(C)** in ONBs. PCNA is highly expressed **(E)** in normoxia, decreased **(F)** in hypoxia, and recovered **(G)** in ONBs. **(I)** Phosphorylated p53 and **(M)** cleaved Caspase-3 are expressed in dental epithelium, including enamel knot, shown in increase **(J,N)** in hypoxia, but in decrease **(K,O)** in ONBs. **(D,H,L,P)** Quantification of labeled intensity of HIF-1α, PCNA, p53, cleaved Caspase-3 in normoxia, hypoxia, ONBs (*N* = 5) (Scale bar = 100 μm. Nor, normoxia; Hyp, Hypoxia; ONBs, Oxygen Nanobubbles; **p-values < 0.01, ***p-values < 0.001. Arrow; positive in enamel knot).

To investigate the induction of hypoxic response, immunofluorescence was performed. At the cap stage (E13), no HIF-1α was found in the tooth germs under normoxic conditions (Figure 2A). However, HIF-1α was induced in the oral epithelium, dental epithelium, and dental mesenchyme under hypoxic conditions (Figure 2B). Interestingly, HIF-1α expression disappeared in the ONB group (Figures 2C,D).

**Figure 3:**
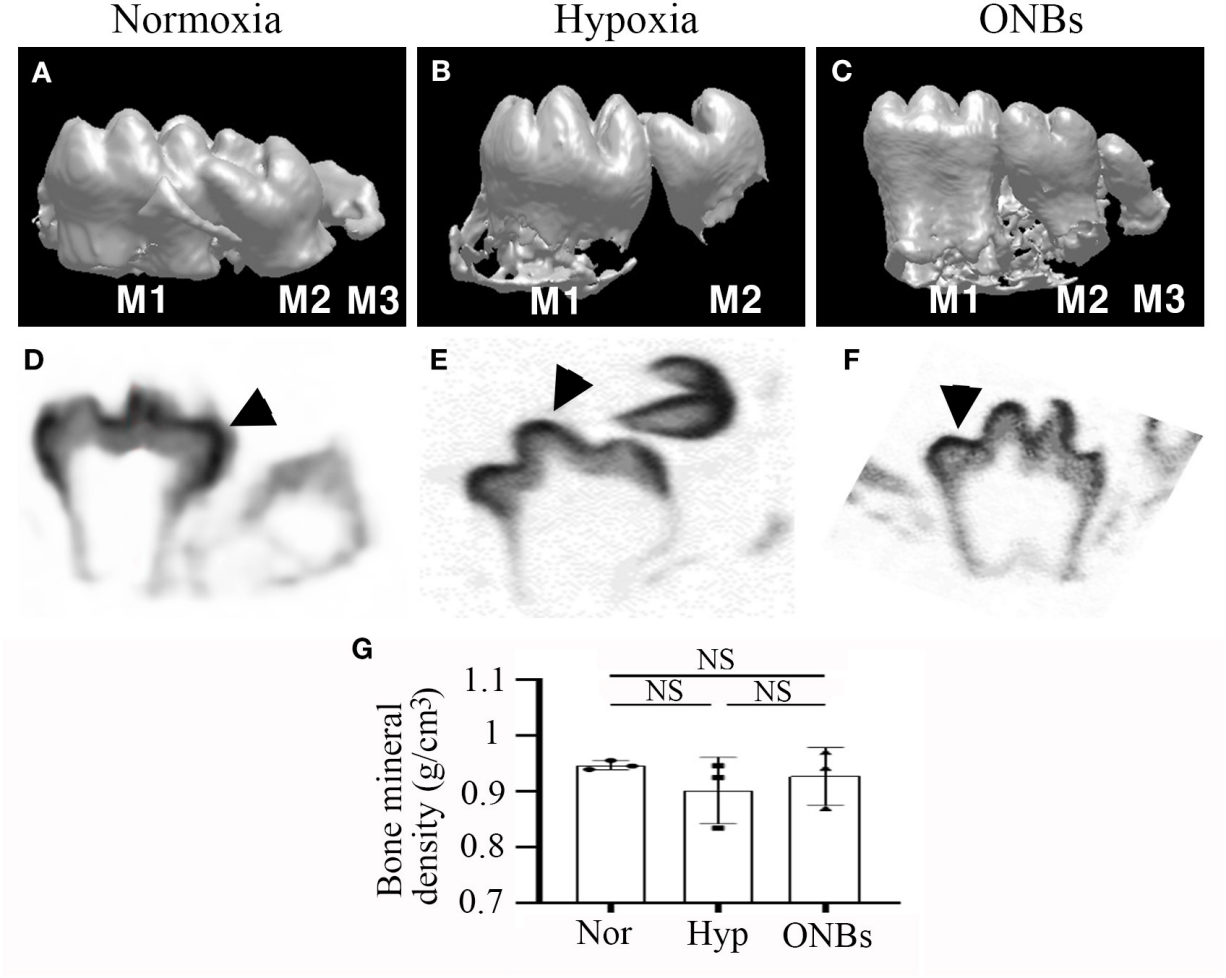
microCT images of Calcified tooth in hypoxia at E13. microCT images of calcified E13 tooth germs in **(A,D)** normoxia, **(B,E)** hypoxia, and **(C,F)** ONBs (*N* = 5). **(A–C)** 3D reconstruction images. **(G)** Quantification of bone mineral density (*N* = 3). **(D–F)** Sagittal view (M, Molar; Arrowheads, mineralization region; NS, non-significant).

To evaluate the changes in cell proliferation and apoptosis under hypoxic conditions, the expression of proliferating cell nuclear antigen (PCNA), phosphorylated p53, and cleaved Caspase-3 were analyzed by immunofluorescence. Many proliferative cells were observed in E13 tooth germs under normoxic conditions (Figure 2E). However, no PCNA positive cells were observed in E13 tooth germs under hypoxic conditions (Figure 2F). Inhibition of cell proliferation caused by hypoxia was restored by treatment with ONBs (Figures 2G,H). Moreover, tooth germs in normoxia underwent apoptosis in the small part of dental epithelium, including enamel knot, as evidenced by expression of phosphorylated p53 and cleaved Capsase-3 (Figures 2I,M). However, many phosphorylated p53-apoptotic cells were found in the dental epithelium and mesenchyme in tooth germs under hypoxic conditions (Figure 2J). Further, cleaved Caspase-3 was detected, especially in the dental epithelium, and in a few cells in the dental mesenchyme (Figure 2N). Interestingly, phosphorylated p53 and cleaved Caspase-3 positive cells were rarely found in tooth germs treated with ONBs (Figures 2K,L,O,P). However, p53 expression was not shown in each condition (data not shown). To investigate the effect of oxygen on the mineralization of tooth, E13 tooth germs were cultured *in vitro* in normoxia, hypoxia for 3 h, and with ONBs for another 1 h, followed by transplantation of these culture tooth germs into kidney capsule for 4 weeks for calcification (Figure 3). Compared to the mineralization of teeth under normoxic conditions (Figures 3A,D), the mineralization of E13 tooth germs cultured under hypoxic conditions (Figures 3B,E) or treated with nanobubbles were not significantly different (Figures 3C,F,G).

### Induction of HIF-1α and Change in Proliferation and Apoptosis in Tooth Germs at E16 Under Hypoxic Conditions

**Figure 4:**
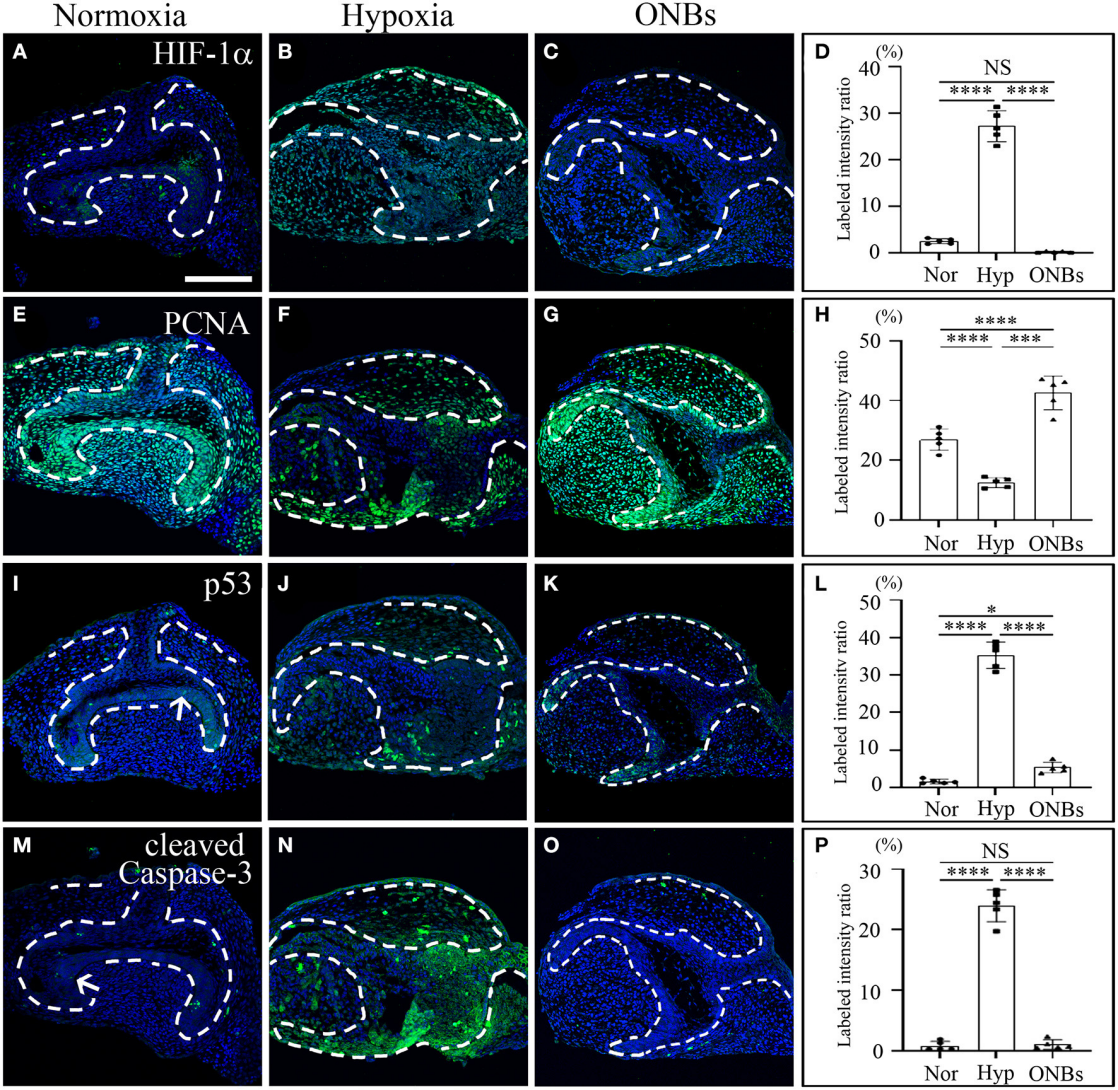
Change of cell proliferation and apoptosis at E16 tooth germs in normoxia, hypoxia, and ONB treatment. Bell stage tooth germs (E16) cultured in normoxia, hypoxia, ONBs were compared. Immunofluorescence was performed to show the expression change of **(A–C)** HIF-1α, **(E–G)** PCNA, **(I–K)** phosphorylated p53, **(M–O)** cleaved Caspase-3 in the frontal sections of tooth germs. HIF-1α expression is not expressed **(A)** in normoxia, increased **(B)** in hypoxia, but decreased **(C)** in ONBs. PCNA is highly expressed **(E)** in normoxia, decreased **(F)** in hypoxia, and recovered **(G)** in ONBs. Phosphorylated p53 and cleaved Caspase-3 are expressed little at enamel knot **(I,M)** in normoxia, shown in increase **(J,N)** in hypoxia, but in decrease **(K,O)** in ONBs. **(D,H,L,P)** Quantification of labeled intensity of HIF-1α, PCNA, phosphorylated p53, cleaved Caspase-3 in normoxia, hypoxia, ONBs (*N* = 5) (Scale bar = 100 μm. Nor, normoxia; Hyp, Hypoxia; ONBs, Oxygen Nanobubbles; *p-values < 0.05, ***p-values < 0.001, ****p-values < 0.0001, Arrow; positive cells in enamel knot).

**Figure 5:**
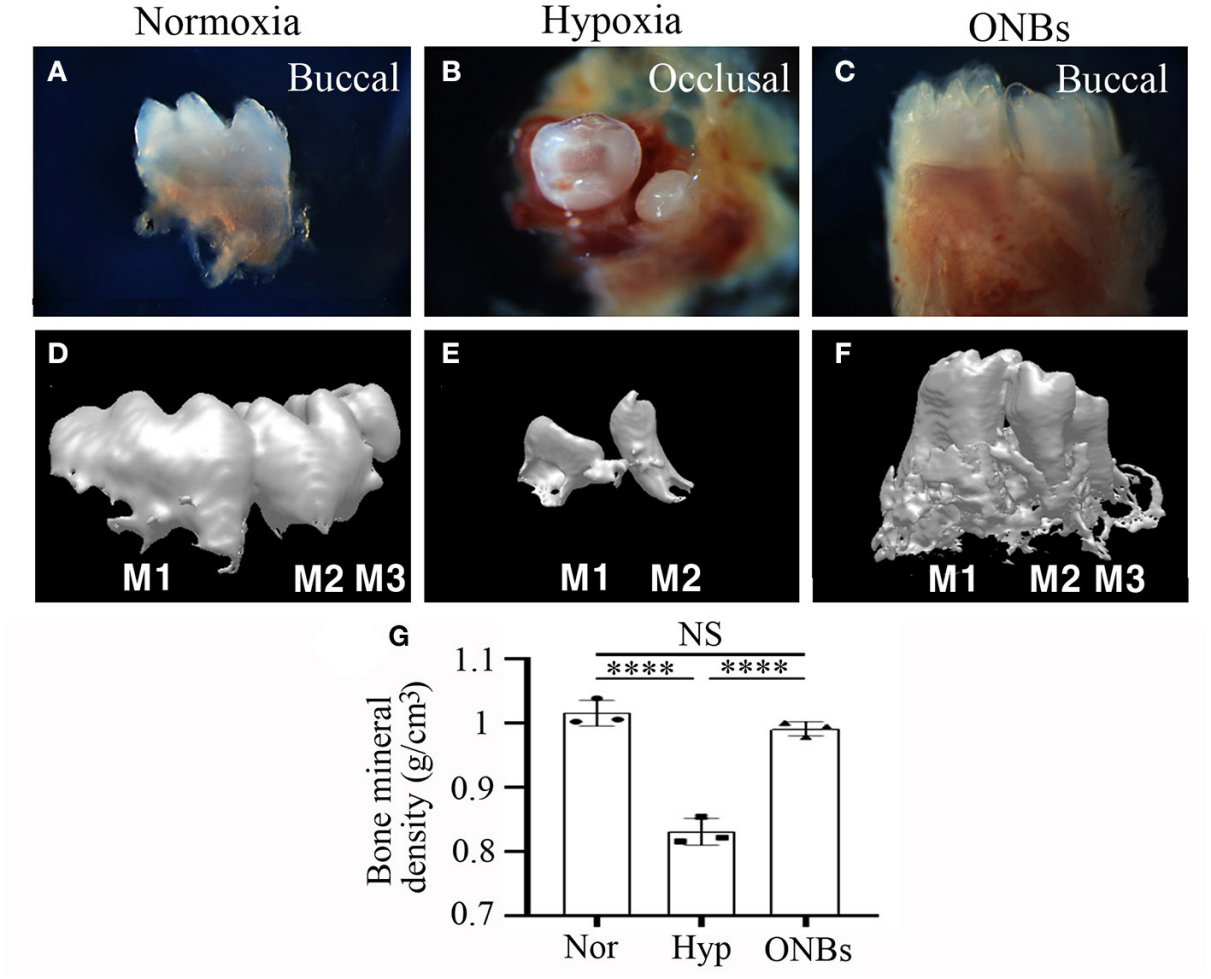
Images of Calcified tooth in hypoxia at E16. **(A–C)** Images and **(D–F)** 3D reconstruction images of micro-CT of calcified E16 tooth germs in **(A,D)** normoxia, **(B,E)** hypoxia, and **(C,F)** ONBs (*N* = 5). **(G)** Quantification of bone mineral density (*N* = 3) (**A,C,D–F** are buccal view. **B** is occlusal view). ****p-values < 0.0001.

Furthermore, immunofluorescence was performed to evaluate the changes in cell proliferation and apoptosis under hypoxic conditions at the bell stage. At the bell stage (E16), few HIF-1α positive cells were found in the dental epithelium and mesenchyme in normoxia (Figure 4A). However, hypoxia-induced HIF-1α were found in the dental epithelium and mesenchyme (Figure 4B). In ONBs, no HIF-1α positive cells were observed in the tooth germs (Figures 4C,D). Thus, ONBs reversed the hypoxic response in the tooth germs not only at the cap stage but also at the bell stage. PCNA was expressed in the dental epithelium and mesenchyme under normoxic conditions (Figure 4E), whereas its expression decreased significantly in the tooth germs under hypoxic conditions (Figure 4F). The reduced cell proliferation was restored by ONBs (Figures 4G,H). Hypoxia-induced apoptosis in E16 was very similar to that observed in E13 (Figure 2). Phosphorylated p53 and cleaved Caspase-3, markers of cell apoptosis, were expressed in very few cells in the tooth epithelium including enamel knot and mesenchyme at E16 under normoxic conditions (Figures 4I,M). However, in tooth germs under hypoxic conditions, the number of phosphorylated p53 and cleaved Caspase-3 positive cells in the tooth epithelium and mesenchyme at E16 were greatly increased (Figures 4J,N), and the numbers were restored following ONBs treatment (Figures 4K,L,O,P). These results suggest that ONB reverses the hypoxic response of tooth germs at the cap and bell stages. Furthermore, cell proliferation and apoptosis induced by hypoxic conditions were restored by ONBs in the developing tooth germs. The mineralization of E16 tooth germs was different from that of E13 as evident from the micro-CT images (Figure 5). Micro-CT revealed that tooth size was smaller under hypoxic conditions (Figures 5B,E) than that under normoxic conditions (Figures 5A,D). Further, only 1 or 2 teeth were observed in hypoxic condition (Figures 5B,E), whereas three teeth, including M1, M2, and M3 were found in normoxic condition. Furthermore, the cusp morphology of the tooth was flattened in hypoxic conditions. Following ONBs treatment, three teeth were observed, and the teeth sizes were similar to that of the normoxic tooth (Figures 5C,F). The mineralization density of teeth was decreased by hypoxic condition than by normoxia condition, but this decreased mineralization was reversed with ONBs (Figure 5G).

To verify that tooth germs under hypoxia recovered with ONBs, the tooth germs were cultured under hypoxic conditions for 3, 6, 9, and 18 h, respectively. However, tooth germs treated with hypoxia for longer than 3 h failed to recover even with ONBs. Therefore, in this study, hypoxia treatment for 3 h was carried out in the experimental group (data not shown).

### Effects of Reduced Oxygen Level on Energy Metabolism in Tooth Germs at E13 and E16

**Figure 6:**
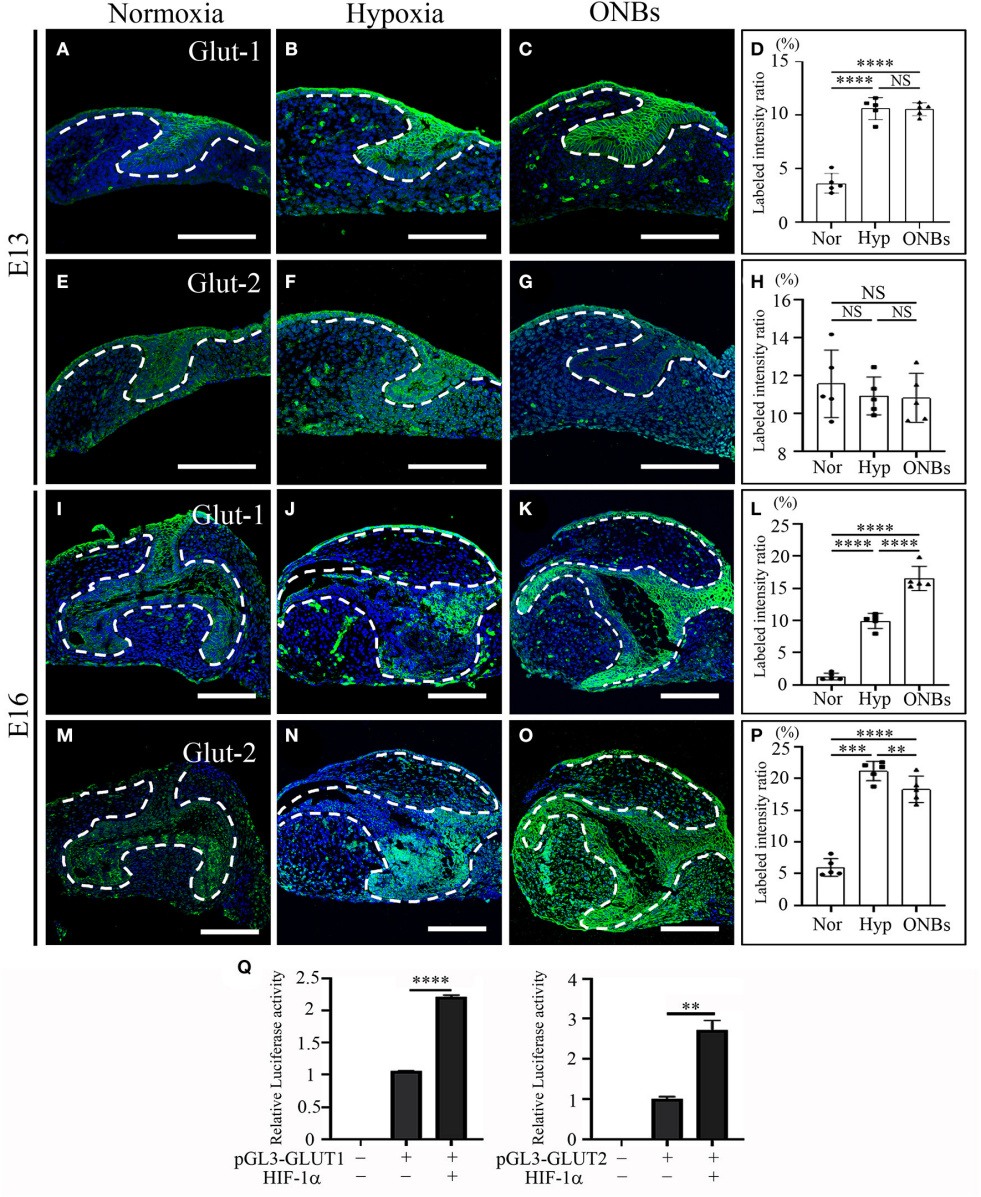
Change of Glucose transporters such Glut-1, and Glut-2 at E13 and E16 in normoxia, hypoxia, and ONB treatment. Cap stage tooth germs (E13) and bell stage tooth germs (E16) cultured in normoxia, hypoxia, ONBs were compared. The expression of Glut-1 at E13 **(A)** in normoxia, **(B)** in hypoxia, and **(C)** in ONBs. **(D)** Glut-1 expression in hypoxia shows in increase than that in normoxia, and similar as ONBs. Glut-2 expression are similar among **(E)** in normoxia, **(F)** hypoxia, and **(G)** ONBs. The expression of Glut-1 at E16 **(I)** in normoxia, **(J)** in hypoxia, and **(K)** in ONBs. At E16, Glut-1 expression **(J)** in hypoxia shows in increase than that **(I)** in normoxia, and lower than **(K)** in ONBs. Glut-2 expression **(N)** in hypoxia is stronger than **(M)** in normoxia. **(O)** When ONBs treatment, the expression of Glut-2 is slightly reduced compare to hypoxia. These results were quantified in **(D,H,L,P)** (*N* = 5). **(Q)** Compared to the pGL3-basic, treatment with 3 μg of HIF-1α expression vector leads to a significant increase of GLUT-1 and GLUT-2 containing HIF-1α binding site (*N* = 3) (Scale bar = 100 μm. Nor, normoxia; Hyp, Hypoxia; ONBs, Oxygen Nanobubbles; **p-values < 0.01, ***p-values < 0.001, ****p-values < 0.0001).

To determine the effect of hypoxia on metabolism, we examined Glut-1 and Glut-2 expression under hypoxic conditions, given that Glut-1 and Glut-2 are expressed during tooth development (Ida-Yonemochi et al., 2012). Glut-1 expression was especially shown in the dental epithelium under normoxic conditions at E13 (Figure 6A). Under hypoxic conditions, Glut-1 expression increased in both the dental epithelium and dental mesenchyme (Figure 6B). Interestingly, hypoxia-induced Glut-1 expression was not much increased in tooth germs following ONBs treatment (Figures 6C,D). Glut-2 was expressed mainly in the dental epithelium under normoxic conditions but expression was weaker than that of Glut-1 (Figure 6E). Under hypoxic conditions, Glut-2 expression was not significantly induced in the dental mesenchyme as well as in the dental epithelium (Figure 6F). Furthermore, ONBs treatment did not change Glut-2 expression (Figures 6G,H). At E16, Glut-1 was expressed in the dental epithelium and slightly expressed in the dental mesenchyme under normoxic conditions (Figure 6I). Under hypoxic conditions, Glut-1 expression increased (Figure 6J), and the expression increased further in the dental epithelium and dental mesenchyme with ONBs treatment (Figures 6K,L). Glut-2 was expressed in the dental epithelium, and was slightly expressed in the dental mesenchyme under normoxic conditions (Figure 6M). Glut-2 expression increased in the dental epithelium under hypoxic conditions (Figure 6N). With ONBs, Glut-2 expression was slightly decreased than under hypoxic condition, but increased than under normoxic condition (Figures 6O,P). HIF-1α modulates GLUT-1 and GLUT-2 promoter activity. As shown in Figure 6Q, GLUT-1 and GLUT-2 promoter activity was significantly increased following transient expression of HIF-1α. These results suggested that HIF-1α by hypoxic conditions during tooth development induced glucose metabolism by positively regulating Glut-1 and Glut-2. Moreover, glucose metabolism, including Glut-1 and Glut-2 expression, is necessary for the repair of tooth mineralization at E16 by oxygen.

### Decreased Bmps and Shh Expression Under Hypoxic Tooth Germs

**Figure 7:**
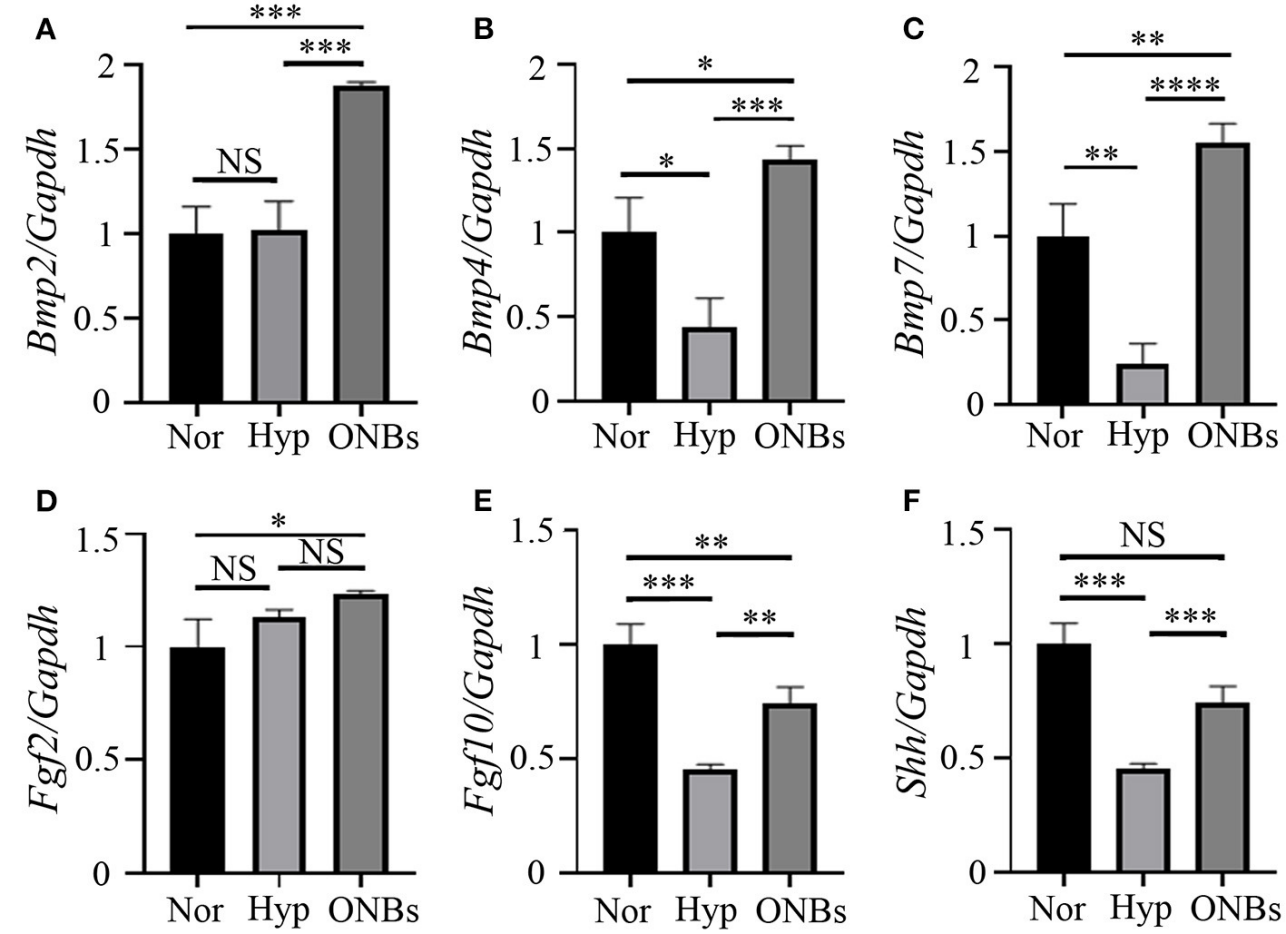
Signaling pathways related with hypoxic condition Expression analysis of Bmp signaling, Fgf signaling, and Shh signaling with tooth germs under normoxia, hypoxia, and ONBs condition. **(A)** *Bmp2* expression levels in tooth germs under normoxia is not changed with hypoxia condition, but upregulated with ONBs. The expression level of **(B)** *Bmp4* and **(C)** *Bmp7* was downregulated in tooth germs under hypoxic condition, but restored with ONB treatment. **(D)** *Fgf2* expression is not changed with hypoxic condition, and ONBs. However, **(E)** *Fgf10* expression level is decreased in tooth germs under hypoxic condition, and shows in increase of tooth germs with ONB treatment. **(F)** *Shh* expression is downregulated under hypoxic condition, and this down regulated expression is recovered with ONBs (Expression level is normalized with *Galdh*. *p-values < 0.1, **p-values < 0.01, ***p-values < 0.001, ****p-values < 0.0001).

In present study, we performed qRT-PCR to identify signaling pathways associated with the hypoxic conditioned tooth germs. Tooth morphogenesis are regulated by epithelial-mesenchymal interactions, which are mediated by signaling pathways including Hedgehog (Hh), Fibroblast growth factor (FGF), and Bone morphogenic protein (Bmp) (Jussila and Thesleff, 2012; Graf et al., 2016). The expression levels of *Bmp2, Bmp4*, and *Bmp7*, which are related Bmp genes with tooth development (Graf et al., 2016), were checked. The expression level of *Bmp2* were not changed under hypoxic condition but increased with ONBs (Figure 7A). On the other hand, the expression level of *Bmp4* (Figure 7B) and *Bmp7* (Figure 7C), which have been known to be expressed in enamel knot and dental mesenchyme (Graf et al., 2016), was decreased under hypoxic condition, and the expression was significantly increased with ONB-restored tooth germs. *Fgf2*, which plays a role for amelogenesis, expression was not regulated with hypoxia (Du et al., 2018). Furthermore, the expression was not changed with ONBs, neither (Figure 7D). During tooth development, *Fgf10* is expressed in dental mesenchyme during the early stage, and in the differentiating odontoblasts at later stage (Du et al., 2018). The expression level of *Fgf10* was downregulated with hypoxic condition, and the expression level was increased with ONBs (Figure 7E). *Shh* is involved in tooth development, and expressed in enamel knot and in dental epithelium (Cho et al., 2011). Hypoxic tooth germs downregulated Shh expression, while ONBs restored the expression level of *Shh* (Figure 7F). These results suggested that oxygen level regulates tooth-related signaling pathway, and mineralization of tooth development.

### Characteristic Defects in Enamel and Dentin in Hypoxic Tooth Germs Are Rescued With ONBs

**Figure 8:**
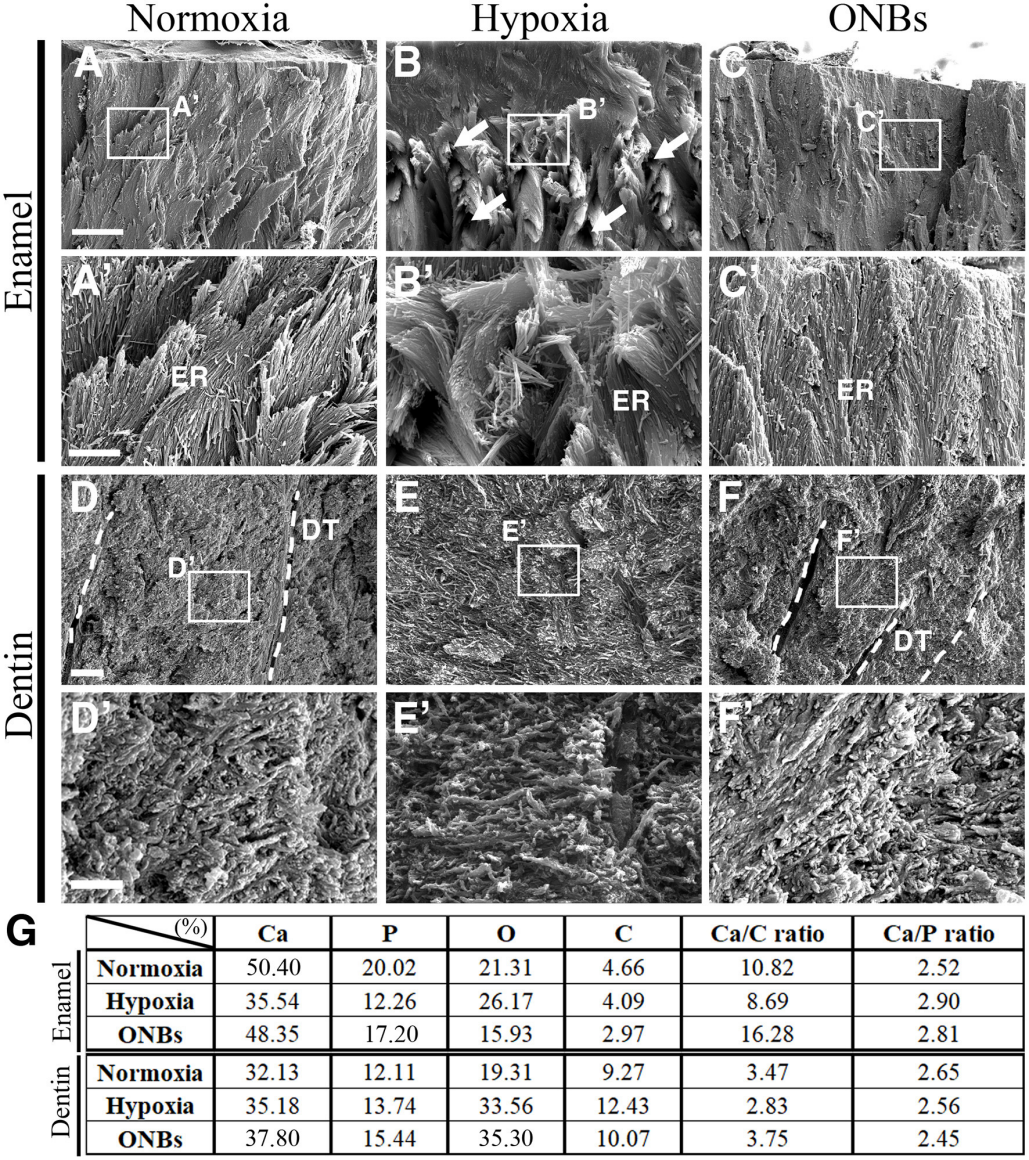
SEM images of calcified teeth with Hypoxic condition SEM images of **(A–C)** enamel and **(D–F)** dentin of cut surface from the cuspal parts of calcified teeth in normoxia, hypoxia, and ONBs. **(A,A')** In normoxia teeth, enamel rods are well-organized. **(B,B')** Very disorganized enamel rods in teeth in hypoxia. **(C,C')** Enamel rods are recovered to well-organized. **(E,E')** Dentin in teeth in hypoxia condition has no significant change in structure compared to **(D,D')** dentin in teeth in normoxia teeth, but is very sparse and has many pores. **(F,F')** The dentin of the teeth with ONBs treatment recovered to be denser and more compact than that of normoxia. **(G)** EDS analysis of calcified teeth under hypoxia (*N* = 3) [Scale bar = 10 μm **(A–C)**, 2 μm **(A'–C',D–F)**, 600 nm **(D'–F')**, **(A'–F')** are higher magnification in **(A–F)**, dotted line = dentinal tubules (DT); ER, enamel rods].

To analyse the hypomineralization defects in enamels of hypoxic tooth germs, we examined the enamel structure of calcified teeth under hypoxic conditions and ONBs treatment using scanning electron microscopy (SEM) (Figure 8). The enamel of teeth in normoxia showed a solid structure as enamel rods (Figures 8A,A'), whereas the enamel rods of teeth under hypoxic conditions were disorganized, and many pores were observed (Figures 8B,B'). The teeth treated with ONBs had enamel structures similar to those of normoxic teeth (Figures 8C,C'). The surface characteristics of the teeth under normoxic conditions indicated a compact and mineralized dentin (Figures 8D,D'). Compared to the teeth under normoxic conditions, dentinal tubules were not clearly seen in the porous and unclear dentin surface of the teeth under hypoxic conditions (Figures 8E,E'). Crystallization was very low under hypoxic conditions compared to that under normoxic conditions. The unmineralized dentin observed under hypoxic conditions was totally rescued by ONBs (Figures 8F,F').

Quantitative analysis of the enamel and dentin using energy dispersive spectroscopy (EDS) revealed that both the enamel and dentin were mainly composed of Ca, P, O, and C under normoxic conditions (Figure 8G). The EDS patterns of teeth under hypoxic conditions were different from those of teeth under normoxic conditions (Supplementary Figure 1). Ca and P levels were decreased in the enamel of teeth under hypoxic conditions, whereas the elements O and C were similar to the elements in the tooth under normoxic conditions. Levels of Ca, P, O, and C were increased in the dentin of teeth under hypoxic conditions, compared to the elements in the teeth in normoxic conditions. After ONB treatment, Ca and P were restored in the enamel, but O and C levels were decreased compared to that in normoxia and hypoxia. After ONBs treatment, Ca, P, and O were increased in the dentin more than that of the dentin in normoxic and hypoxic conditions, whereas the level of C decreased to the normoxia levels. These results suggested that hypomineralization of the enamel and dentin occurred under hypoxic conditions, and treatment with ONBs can rescue the hypomineralization.

### Effect on Tooth Mineralization Under Hypoxic Condition at E16

**Figure 9:**
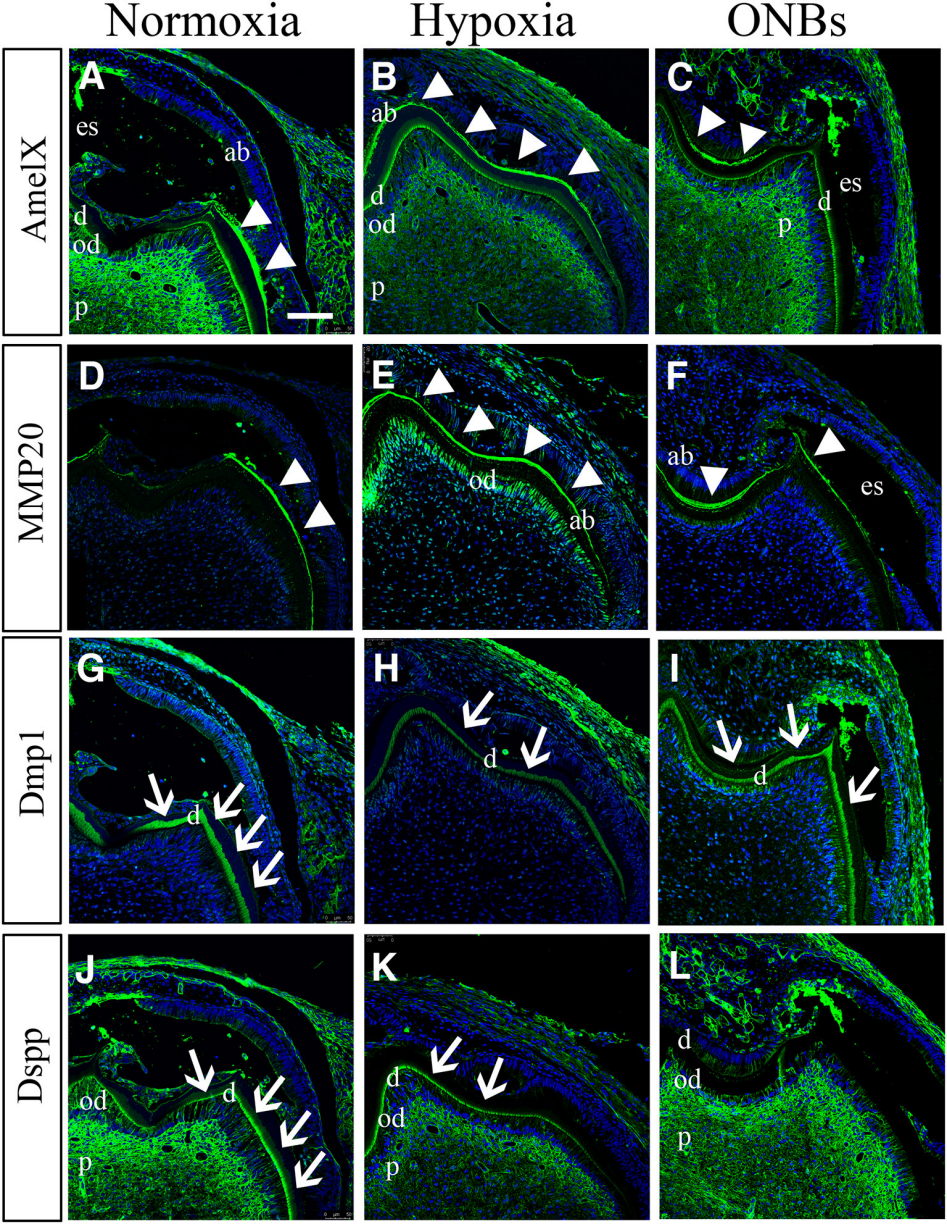
Effect on tooth calcification under hypoxic condition at E16. E16 tooth germs were transplanted into kidney capsule for the calcification. **(A,D)** Under normoxic conditions, enamel space by enamel decalcification, shrink ameloblasts, dentin, odontoblasts, and pulp are found. **(A)** AmelX and **(D)** MMP20 is strongly detected in the enamel matrix (arrowheads) in the secretory ameloblasts. **(B,D)** In hypoxia condition, ameloblasts are obviously longer compared with normoxia tooth. **(B)** AmelX and **(E)** MMP20 expression are shown in the all area of the tooth enamel organ. **(C,F)** With ONBs, ameloblasts are shorten, similar to normoxia tooth. **(C)** AmelX and **(D)** MMP20 is expressed in the enamel matrix in the secretory ameloblasts. Under normoxia condition, **(G)** Dmp1 is expressed in dentin (arrow), and **(J)** DSPP are expressed in odontoblasts, and in dentin (arrow). In hypoxia, **(H)** Dmp1 is expressed very weakly in dentin, and **(K)** DSPP expression is shown weak in dentin and odontoblasts. With ONBs, **(I)** Dmp1 and **(L)** DSP are very strongly expressed in dentin, and in odontoblasts (*N* = 3) (Scale bar = 100 μm. ab, ameloblast; es, enamel space; d, dentin; ob, odontoblast; d, dentin; white arrowhead, enamel matrix; arrow, dentin).

To identify the hypomineralization of enamel and dentin under hypoxic conditions, tooth germs were cultured with normoxia, hypoxia, and ONBs condition, incubated for 1 week under the kidney capsule for the calcification, and histological analysis was performed with enamel and dentin proteins (Figure 9). Under normoxic condition, enamel space by enamel decalcification, shrink ameloblasts, dentin, odontoblasts, and pulp were found (Figure 9A). AmelX (Figure 9A) and MMP20 (Figure 9D) was strongly detected in the enamel matrix in the secretory ameloblasts only, but disappeared at the tip of molar cusp which were undergoing enamel mineralization. The enamel layer showed more matrix protein and ameloblasts were obviously longer compared with that in normoxia (Figures 9B,E). Interestingly with ONBs, matured ameloblasts where were processing enamel mineralization, were shorten, similar to normoxia tooth (Figures 9C,F). AmelX and MMP20 expression were found in the enamel matrix in the secretory ameloblasts only (Figures 9C,F). These abnormal expression results demonstrated that in hypoxic condition tooth, enamel crystallization were aberrant, and occurred an imperfecta in amelogenesis.

Furthermore, we confirmed dentinogenesis (Dmp1 and DSPP) using immunofluorescence. Dmp1 and DSPP were expressed in odontoblasts, dentin, and pulp (Figures 9G,J) of the normoxic teeth. However, the expression of Dmp1 and DSPP decreased in the odontoblast layer and dentin in hypoxic teeth (Figures 9H,K). Interestingly, DMP1 and DSPP were expressed very strongly not only in the odontoblast layer but also in the dentin tubules with ONBs (Figures 9I,L). These results revealed that decrease in oxygen level at E16, when mineralization begins, alters the tooth size and cuspal morphology, and results in defects in amelogenesis and dentinogenesis. Furthermore, ONB-mediated increase in oxygen level restores the morphology and ameliorates the defects induced by the hypoxia.

## Discussion

Supply of sufficient oxygen is essential for the survival of tissues, otherwise, cells and tissues undergo necrosis and programmed cell death (Suvarnapathaki et al., 2019). Our findings reveal that it is possible to reverse hypoxic tooth germs with oxygen nanobubbles. The present study identified the effects of change in oxygen level on tooth mineralization during tooth development. Hypoxic condition promote angiogenesis, glucose transport, anaerobic metabolism, invasion, resistance, and survival, particularly by modulating the apoptotic process (Al Tameemi et al., 2019). Numerous reports have shown that HIF-1α is closely linked to positive and negative regulation of the apoptotic process via the p53 or Caspase pathway (Bruick, 2000; Erler et al., 2004; Cosse et al., 2009). Hypoxia has a significant effect on neuronal cell apoptosis, via Caspase-3 activation (Deng et al., 2019). Hypoxia ischemia leads to significant increase in Caspase-3 expression and neuronal apoptosis in the brain of neonatal mice (Deng et al., 2019). In most cases, increase in p53 level induces apoptosis in cells exposed to hypoxia, although an increase in p53 protein level without subsequent apoptosis has been observed (Kilic et al., 2007; Sermeus and Michiels, 2011). The presence of both HIF-1α and p53 seems to be essential for hypoxia-induced cell death (Sermeus and Michiels, 2011). In the present study, exposure of tooth germs at E13 and E16 to hypoxia for 3 h increased cell apoptosis and decreased cell proliferation, as evidenced by that the expression of phosphorylated p53 and cleaved Caspase3 were increased and the expression of PCNA was decreased. The hypoxia-induced effects in E13 and E16 tooth germs were reversed with ONBs. ONBs reverse hypoxia and suppress HIF-1α activity in tumor cells (Khan et al., 2018). Thus, ONB treatment can overcome hypoxic stress caused by cellular processes, including cell proliferation and apoptosis, not only in cancer cells, but also in normal tissues.

A previous study demonstrated that the expression pattern of GLUTs, including Glut-1 and Glut2 during tooth development (Ida-Yonemochi et al., 2012). Glut-1 was expressed in the epithelium of bud to cap stage molar tooth germs, but was decreased in the bell stage. Glut-2 expression appeared at E16 (bell stage). Furthermore, inhibition of Glut-1/2 resulted in a change in tooth morphology. HIF-1α regulates the activity of GLUT-1 and GLUT-3 (Chen et al., 2001; Hayashi et al., 2004; Liu et al., 2009). Furthermore, genetic deletion of Glut-1, either before or after the onset of chondrogenesis in the limb, severely impairs chondrocyte proliferation and hypertrophy, resulting in dramatic shortening and hypomineralization of the limbs (Lee et al., 2018). In this study, using luciferase assay, we demonstrated that HIF-1α positively regulates both Glut-1 and Glut-2. Glut-1 expression was found to increase in the tooth germs under hypoxic conditions at E13 and E16, and was similar or increased further with ONBs. Glut-2 expression was increased in hypoxic condition only at E16, when mineralization begins. When the oxygen level is low at the initial stage of tooth development (soft tissue stage), tooth repair is performed by including glucose metabolism through Glut-1 rather than Glut-2. However, if the oxygen level drops at the beginning of the mineralization during the tooth development process, tooth repair is performed by inducing glucose metabolism through both Glut-1 and Glut-2.

Tooth morphogenesis is regulated by the dental epithelium, which differentiates to enamel producing ameloblasts and the growth and folding of inner dental epithelium determines the size and morphology of tooth crown (Jussila and Thesleff, 2012). Furthermore, the epithelial signaling centers expressing multiple signaling molecules, such as primary enamel knot, and secondary enamel knots plays a pivotal role for the tooth morphogenesis regulated with the mesenchyme (Sadier et al., 2019) and initiate differentiation of odontoblasts (Thesleff et al., 2001). After the odontoblasts differentiation, they signal back to the dental epithelium, which is involved in the ameloblast induction include *Bmp2*, and *Bmp4* (Wang et al., 2004). Furthermore, Shh from the dental epithelial cells is required to ameloblast differentiation and maturation (Dassule et al., 2000). Fgf signaling is known to be important in the differentiation of ameloblasts. *Fgf2* and *Fgf10* regulates enamel and dentine formation in mouse tooth germ (Harada et al., 2002; Tsuboi et al., 2003). In addition, many studies reported that Bmp signaling, Fgf signaling, Shh signaling is regulated by hypoxia (Conte et al., 2008; Wang et al., 2010; Wu and Paulson, 2010; Zhang et al., 2018). When tooth germs undergo the hypoxic condition during the mineralization, tooth morphology and mineralization have defects through cell proliferation, apoptosis and glucose metabolism changes. It was confirmed that the expression of *Bmp4* and *Bmp7, Fgf10*, and *Shh were* decreased in the low oxygen condition. When ONB was treated, the expression of *Fgf10* and *Shh* was reversed. Furthermore, the expression level of *Bmp4* and *Bmp7* was reversed, which was much higher than that in the normoxia condition. Therefore, cellular events due to oxygen changes interact with Bmp, Shh, and Fgf signaling pathway to regulate the size and morphology of tooth, as well as amelogenesis and dentinogenesis.

Enamel development consists of secretory and maturation stage (Lagerstrom-Fermer and Landegren, 1995). During secretory stage, columnar ameloblasts secrete enamel proteins, such as amelogenin (AmelX), ameloblastin, and enamelin, into the enamel matrix (Lagerstrom-Fermer and Landegren, 1995). MMP20 expressed in ameloblasts at the secretory stage have the enamel proteins (Bartlett et al., 2006). During maturation stage, ameloblasts shrink in size and reduce the expression of enamel proteins, which undergo enzymatic degradation by Kallikrein-related peptidase-1 (KLK-4) to facilitate enamel mineralization (Smith et al., 2017). When hypoxic teeth were incubated for 1 week in kidney capsules, amelogenesis appeared to be delayed due to the remaining high expression of enamel matrix, long ameloblasts, and enamel protein. Eventually, after 4 weeks, the mineral density decreased by nearly 20%. In addition, dentin mineralization is controlled by several molecular events, such as DSPP, DMP1, however, several mutations in the Dspp gene results in widened predentin zoes, decreased dentin width, and high incidence of pulp exposures similar to that in DGI (Sreenath et al., 2003). With hypoxic condition when mineralization starts, Dspp and Dmp1 expression is decreased than that in normoxia after 1-week calcification. After 4 weeks of calcification, AmelX-positive cells in matured ameloblasts were rarely found in hypoxic calcified teeth, and MMP20-positive ameloblasts in the secretory stage had expression along the DEJ (Supplementary Figures 2B,E). However, ONB treatment showed an increase in AmelX-expressing matured ameloblast and a decrease in MMP20 expression in secretory stage, restoring ameloblast maturation and mineralization in enamel formation (Supplementary Figures 2C,F). Furthermore, DMP1 and DSPP expression was decreased in dentin and odontoblast in hypoxic condition (Supplementary Figures 2H,K) compared to normoxia condition (Supplementary Figures 2G,J). With ONBs, hypomineralization of dentin by hypoxia is restored (Supplementary Figures 2I,L). In addition, Dmp1 and DSPP expression was shown strongly an increase more than that in normoxia. Further study is needed to identify how oversupply of oxygen affects the mineralization of enamel and dentin.

**Figure 10:**
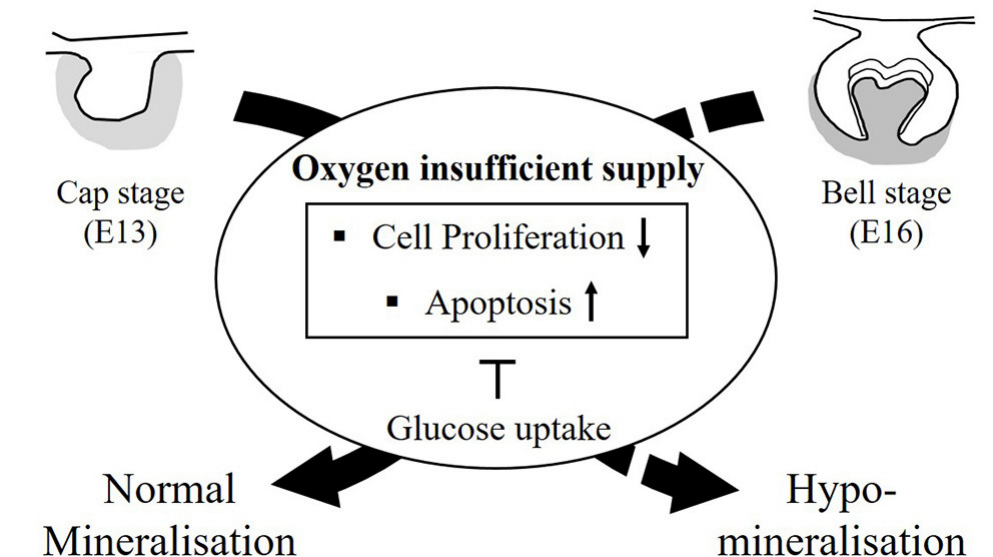
The summary diagram. The tooth germs at the cap stage (E13, all soft tissue) and at the bell stage (E16, start to form hard tissue) were cultured with oxygen insufficient supply, such as hypoxia condition. After 3 h, cell cycle arrest by p53 and apoptosis by Caspase-3 were increased in damaged tooth germ by induced HIF-1α in hypoxic condition. Cell proliferation in damaged tooth germs were decreased. Furthermore, glucose uptake, such as Glut-1, Glut-2 in hypoxic condition was increased for block the cell cycle arrest and apoptosis. The hard tissue, including enamel and dentin is formed in kidney capsule for 4 weeks. Enamel mineralization from tooth germs at E13 (all soft tissue) with hypoxic condition are normally formed. However, the damaged tooth germs with hypoxic condition at E16 (mineralization period) did not produce the normal mineralized teeth, but hypomineralized teeth. The newly developed oxygen nanobubbles can reverse the tooth that has suffered hypoxic damage, and furthermore, this treated tooth with oxygen nanobubbles restore the mineralization.

MIH occurs when problems arise during the mineralization period of the permanent first molar and incisors (Lee et al., 2020). In humans, the mineralization of these teeth begins at the end of the gestation period (Tourino et al., 2016). It has been suggested that MIH occurs in children due to lack of oxygen, and, hypoxic conditions, due to asthma and/or bronchitis during the mineralization period, which may be closely related to amelogenesis. In hypomineralized enamel, the enamel mineral content is reduced, and it exhibits lower hardness values. In the ultrastructure of hypomineralized enamel, the crystals appear to be more loosely and irregularly packed than in the normal enamel (Bozal et al., 2015). Furthermore, the elements of O, Ca, and the Ca/P ratio are not different from those in normal teeth (Martinovic et al., 2015). However, C was significantly increased in hypomineralized enamel compared to that in normal enamel, while the P and Ca/C ratios were significantly lower in hypomineralized enamel than in normal enamel. MIH affects all hard dental tissues, such as enamel and dentin (Padavala and Sukumaran, 2018). In SEM analyses, no structural differences could be discerned between the dentin below normal enamel and the dentin below hypomineralized enamel (Heijs et al., 2007). The level of C was highest in dentin below hypomineralized enamel and lowest in dentin below normal enamel. The Ca/P ratios for dentin below hypomineralized enamel were in principle identical to those of normal enamel. When the Ca/C ratio was analyzed, dentin below hypomineralized enamel had the lowest value, whereas dentin below normal enamel had the highest. In this study, EDS analysis of the enamel revealed that tooth component, such as Ca, P, and Ca/C ratio showed lower under hypoxic condition, but O and Ca/P ratios were very similar. The Ca/C ratio was much lower than that of normoxia tooth. Furthermore, EDS analysis of dentin showed that while the Ca/P ratio was the same as that of normoxia enamel, but the Ca/C ratio was lower than that of dentin under normoxic conditions. The changes of composition of dentin and enamel under hypoxic conditions are similar to those of observed in hypomineralized teeth in MIH. These findings suggest prenatal oxygen deficiency at E16, or bell stage, when the hard tissues, such as enamel and dentin, start to form, as an etiology for hypomineralized teeth through cellular events with glucose metabolism (Figure 10). It was identified that the mineralization defect was restored through ONBs treatment when hypoxia-induced defects occurred for 3 h. However, this study involves restoring embryonic tissue under short exposure to low oxygen conditions. In the future, further studies on efficient oxygen delivery to treat tissues exposed to long-term hypoxia will be needed. In conclusion, we suggest that ONB can be used to successfully restore hypomineralized enamel and dentin caused by hypoxia by supplying oxygen and help researchers develop regenerative medicine treatment to improve clinical care.

## Data Availability Statement

The original contributions presented in the study are included in the article/Supplementary Material, further inquiries can be directed to the corresponding author/s.

## Ethics Statement

The animal study was reviewed and approved by the Yonsei University College of Dentistry, Intramural Animal Use and Care Committee (2019-0306).

## Author Contributions

H-SJ and E-JK designed study. E-JK, J-EL, SY, D-JL, and HM performed the research. E-JK, J-EL, SY, D-JL, HM, HI-Y, JC, and H-SJ discussed the data. E-JK, J-EL, HI-Y, and H-SJ analyzed the data. E-JK, J-EL, and H-SJ wrote the paper. All authors contributed to the article and approved the submitted version.

## Conflict of Interest

The authors declare that the research was conducted in the absence of any commercial or financial relationships that could be construed as a potential conflict of interest.

## Supplementary Material

Supplementary Figure 1 The EDS patterns of enamel and dentin. The peak patterns of EDS analysis of **(A,C,E)** enamel and **(B,D,F)** dentin on mineralized tooth with normoxia, hypoxia condition.

Supplementary Figure 2 Effect on tooth calcification under hypoxic condition at E16. E16 tooth germs were transplanted into kidney capsule for 4 weeks for the calcification. Immunofluorescence was performed to show **(A–C)** AmelX, **(D–F)** MMP20, **(G–I)** Dmp1, and **(J–L)** Dspp expressions. **(A)** AmelX is expressed in mature ameloblasts in normoxia, but **(B)** the expression is found very little in hypoxia. **(C)** With ONBs, AmelX expression is mature ameloblasts are recovered. **(D)** MMP20 in secretory ameloblasts is expressed very little; however, **(E)** MMP20-positive secretory stage cells are increased in hypoxic condition. **(F)** MMP20 expression in ONBs are as similar as that in normoxia. **(G)** Dmp1 and **(J)** DSPP are expressed in odontoblasts, and in dentin in normoxia. In hypoxia, very little **(H)** Dmp1 and **(K)** DSPP positive cells are found in odontoblasts. and in dentin, **(I)** DMP1 and **(L)** DSPP are very strongly expressed in dentin, and in odontoblasts (*N* = 3) (Scale bar = 100 μm. ab, ameloblast; es, enamel space; d, dentin; ob, odontoblast; p, predentin; d, dentin; dotted line, dentioenamel junction; arrowheads; ameloblast, arrow: odontoblast).
