# Supplementary figures and images for: Hypoxia-Responsive Oxygen Nanobubbles for Tissues-Targeted Delivery in Developing Tooth Germs
Source: Front Cell Dev Biol. 2021 Feb 15;9:626224. doi: 10.3389/fcell.2021.626224 (PMC7917193; doi:10.3389/fcell.2021.626224)

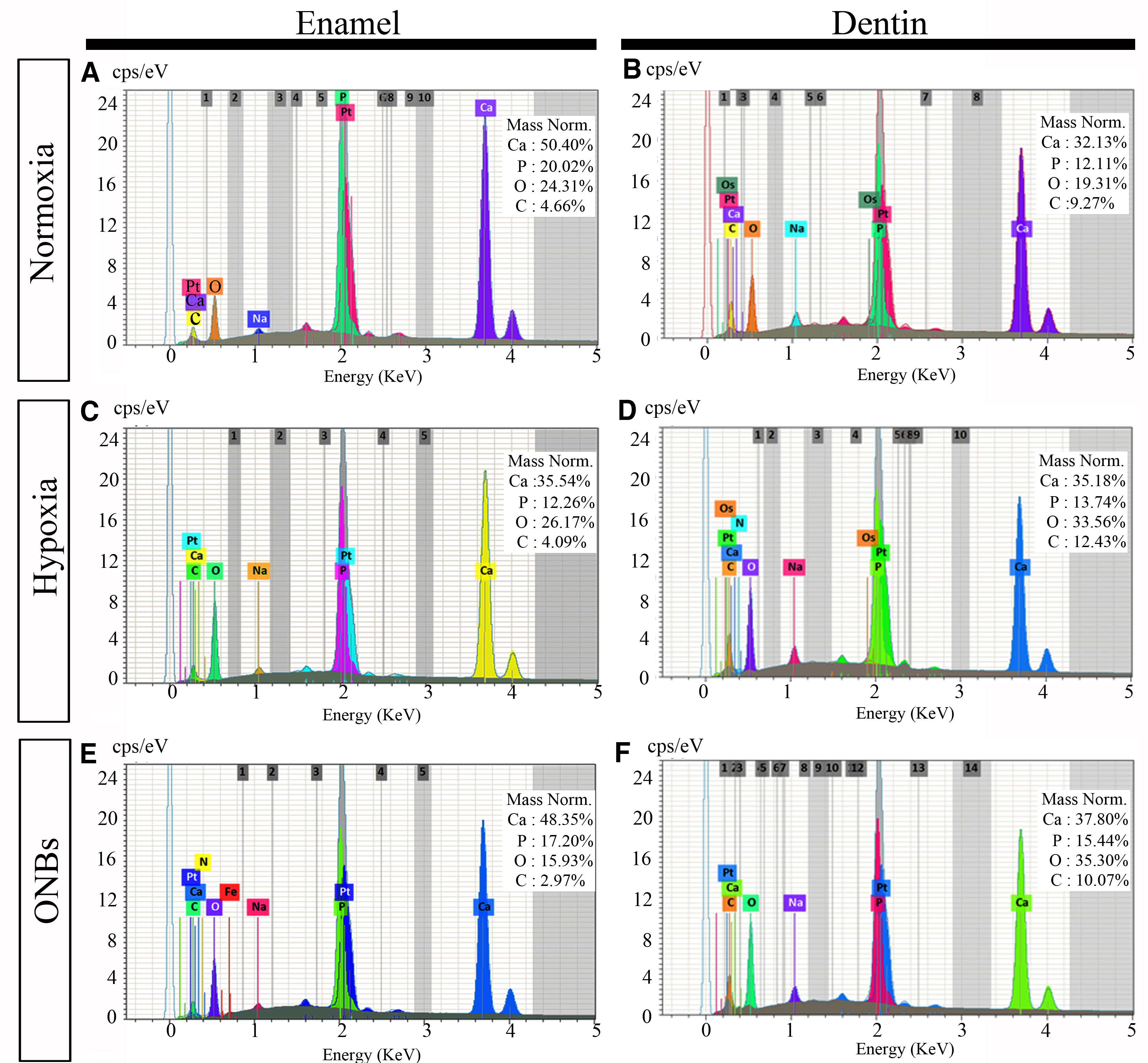

Supplement: Supplementary Figure 1 — The EDS patterns of enamel and dentin. The peak patterns of EDS analysis of (A,C,E) enamel and (B,D,F) dentin on mineralized tooth with normoxia, hypoxia condition. [file Image_1.TIF]

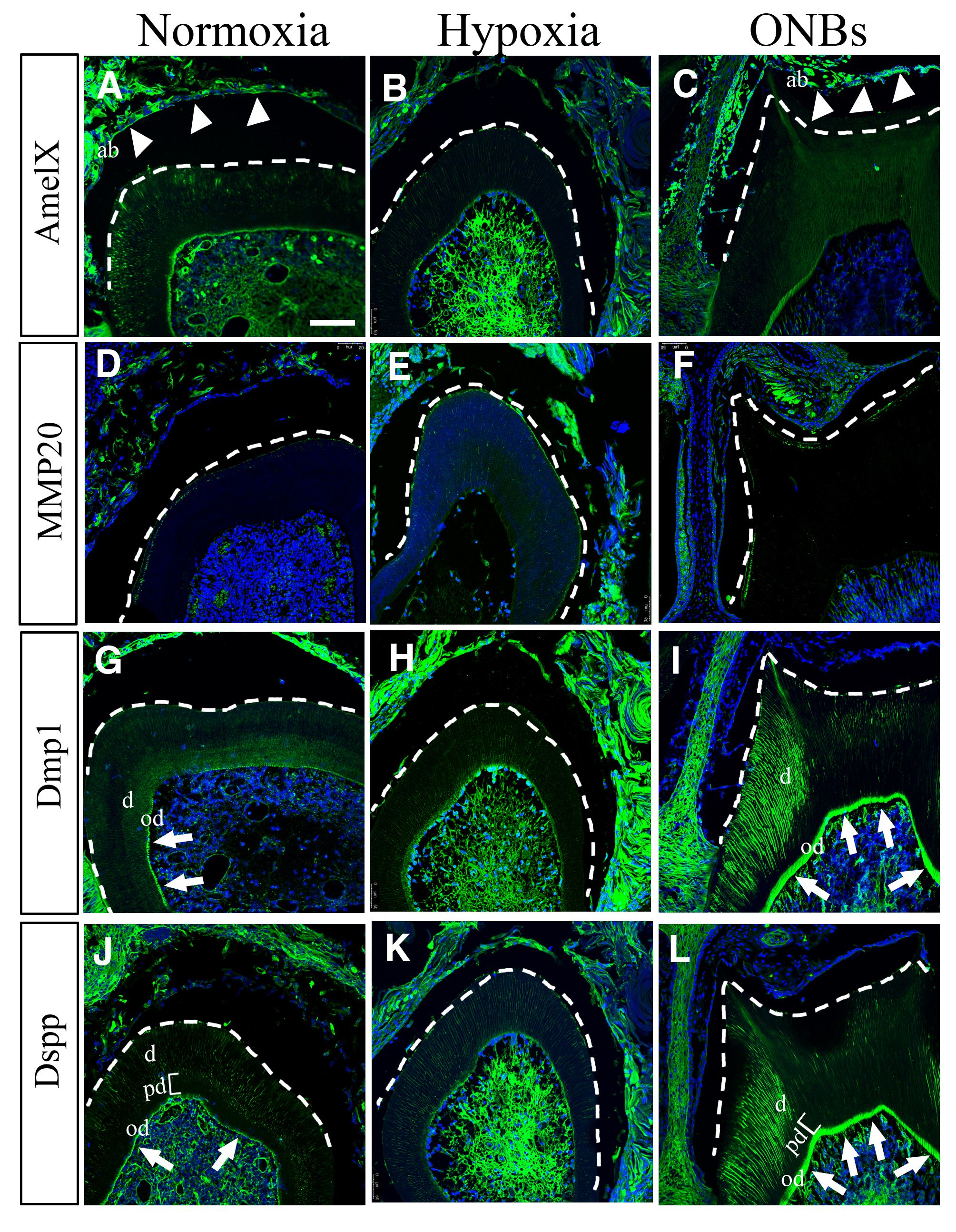

Supplement: Supplementary Figure 2 — Effect on tooth calcification under hypoxic condition at E16. E16 tooth germs were transplanted into kidney capsule for 4 weeks for the calcification. Immunofluorescence was performed to show (A–C) AmelX, (D–F) MMP20, (G–I) Dmp1, and (J–L) Dspp expressions. (A) AmelX is expressed in mature ameloblasts in normoxia, but (B) the expression is found very little in hypoxia. (C) With ONBs, AmelX expression is mature ameloblasts are recovered. (D) MMP20 in secretory ameloblasts is expressed very little; however, (E) MMP20-positive secretory stage cells are increased in hypoxic condition. (F) MMP20 expression in ONBs are as similar as that in normoxia. (G) Dmp1 and (J) DSPP are expressed in odontoblasts, and in dentin in normoxia. In hypoxia, very little (H) Dmp1 and (K) DSPP positive cells are found in odontoblasts. and in dentin, (I) DMP1 and (L) DSPP are very strongly expressed in dentin, and in odontoblasts (N = 3) (Scale bar = 100 μm. ab, ameloblast; es, enamel space; d, dentin; ob, odontoblast; p, predentin; d, dentin; dotted line, dentioenamel junction; arrowheads; ameloblast, arrow: odontoblast). [file Image_2.jpg]
